# Supplementary material for: Prediction of Incident Cancers in the Lifelines Population-Based Cohort
Source: Cancers (Basel). 2021 Apr 28;13(9):2133. doi: 10.3390/cancers13092133 (PMC8125183; doi:10.3390/cancers13092133)
Supplement: Supplementary file 1 [file cancers-13-02133-s001.zip › cancers-1200413-supplementary.pdf]

cancers

MDPI

Article

Prediction of Incident Cancers in the Lifelines Population-Based Cohort

Francisco O. Cortés-Ibañez [1,*], Sunil Belur Nagaraj [2], Ludo Cornelissen [3], Gerjan J. Navis [4], Bert van der Vegt [5], Grigory Sidorenkov [1] and Geertruida H. de Bock [1]

Table S1. Overview of the additional clinical and diet-based variables included/excluded in the study.

	Cancer in follow-up	Without any history of cancer	% of missing values
	n = (4 232)	*n* = (116 188)	
Baseline age (SD)	52.53 (13.12)	43.62 (12.68)	0%
Baseline body mass index (SD)*	26.62 (4.22)	25.97 (4.30)	0%
Baseline alcohol intake grams/day (SD)	8.12 (9.69)	7.20 (8.93)	0%
Smoking packages/year (SD)	10.20 (13.89)	5.90 (9.57)	0%
Baseline physical activity hours/week (SD)	4.51 (5.49)	4.14 (4.80)	0%
Baseline sedentary behaviour TV hours/day (SD)	2.70 (1.53)	2.46 (1.48)	0%
Sex			
Females (%)	2 581 (61.0%)	67 679 (58.2%)	0%
Education level			
Low (%)	1 715 (40.5%)	34 678 (29.8%)	0%
Medium (%)	1 432 (33.8%)	46 238 (39.8%)	0%
High (%)	1 085 (25.6%)	35 272 (30.4%)	0%
Blood test baseline			
Alkaline phosphatase U/L (SD)	64.214 (20.55)	61.98 (17.92) §	67.03%
ALAT U/L (SD)	23.75 (20.59)	23.28 (16.53) §	67.03%
ASAT U/L (SD)	25.04 (12.72)	24.03 (9.83) §	67.03%
Albumin g/L (SD)	44.35 (2.26)	45.01 (2.38) §	67.03%
Calcium mmol/L (SD)	2.28 (0.08)	2.27 (0.08) §	67.03%
Anti- CCP U/mL (SD)	2.33 (15.58)	2.17 (13.71) §	69.42%
CTD screen ratio (SD)	0.28 (0.89)	0.28 (5.38) §	69.42%
Anti-ds DNA U/L (SD)	14. 50 (22.11)	13.31 (21.45) §	99.16%
Phosphate mmol/L (SD)	0.90 (0.16)	0.91 (0.17) §	67.03%
Free T3 pmol/L (SD)	5.17 (0.84)	5.25 (0.80) §	70.12%
Free T4 pmol/L (SD)	15.77 (2.45)	15.81 (2.24) §	70.12%
Gamma-GT U/L (SD)	29.96 (30.65)	26.74 (26.17) §	67.03%
Apolipo A1 HAL1 g/L (SD)	1.53 (0.26)	1.49 (0.25) §	78.92%
Apolipo B100 HALB g/L (SD)	0.94 (0.23)	0.92 (0.24) §	78.92%
hsCRP LCRP mg/L (SD)	2.93 (5.20)	2.55 (4.39) §	68.52%
SSA preg U/mL (SD)	55.66 (174.85)	36.34 (84.86) §	99.16%
TSH mU/L (SD)	2.44 (1.57)	2.56 (4.29) §	70.22%
Ureum mmol/L (SD)	5.44 (1.37)	5.14 (1.27) §	67.03%
Uric acid mmol/L (SD)	0.29 (0.07)	0.29 (0.07) §	67.03%
Basophilic granulocytes 10E9/L (SD)	0.0 (0.02)	0.03 (0.01)	2.23%

Basophilic granulocytes % (SD)	0.54 (0.34)	0.53 (0.32)	2.23%
Creatinine umol/L (SD)	73.67 (1.91)	73.31 (12.99)	0.46%
Cholesterol mmol/L (SD)	5.19 (1.01)	5.06 (1.00)	0.46%
hsCRP.1 mg/L (SD)	3.07 (5.16)	2.17 (3.16) §	68.52%
Eosinophil granulocytes 10E9/L (SD)	0.19 (0.13)	0.18 (0.13)	2.23%
Eosinophil granulocytes % (SD)	3.06 (1.93)	3.07 (1.98)	2.23%
Erythrocytes 10E12/L (SD)	4.67 (0.40)	4.71 (0.39)	0.63%
Glucose mmol/L (SD)	5.16 (0.96)	4.98 (0.82)	0.97%
Neutrophil granulocytes 10E9/L (SD)	3.42 (1.25)	3.30 (1.22)	2.23%
Neutrophil granulocytes % (SD)	54.50 (8.44)	54.07 (8.35)	2.23%
Haemoglobin mmol/L (SD)	8.78 (0.79)	8.73 (0.80)	0.63%
HbA1c mmol/mol (SD)	38.35 (5.33)	36.95 (4.80)	8.19%
HbA1c % (SD)	5.66 (0.49)	5.53 (0.44)	1.06%
Cholesterol HDL mmol/L (SD)	1.50 (0.41)	1.49 (0.39)	0.46%
Haematocrit v/v (SD)	0.42 (0.03)	0.42 (0.03)	0.63%
Potassium mmol/L (SD)	3.92 (0.31)	3.85 (0.31)	0.46%
Cholesterol LDL mmol/L (SD)	3.30 (0.93)	3.21 (0.91)	0.47%
Leukocytes 10E9/L (SD)	6.29 (2.68)	6.07 (1.69)	0.63%
Lymphocytes 10E9/L (SD)	2.04 (0.62)	2.02 (0.59)	2.23%
Lymphocytes % (SD)	33.67 (7.95)	34.19 (7.69)	2.23%
Monocytes 10E9/L (SD)	0.49 (0.16)	0.48 (0.15)	2.23%
Mononuclear cells % (SD)	8.20 (2.05)	8.11 (1.95)	2.23%
Sodium mmol/L (SD)	141.69 (1.97)	141.72 (1.85)§	67.07%
Triglycerides mmol/L (SD)	1.21 (0.72)	1.17 (0.80)	0.46%
Thrombocytes 10E9/L (SD)	252.14 (77.12)	249.34 (54.98)	0.69%
Urine test baseline			
Albumin 24hrs urine mg/L (SD)	10.79 (60.73)	6.26 (42.41) §	59.45%
Creatinine 24hrs urine mmol/L (SD)	7.76 (3.64)	8.52 (4.12)	0.49%
Diet components baseline			
Eggs g/day (SD)	15.00 (14.72)	14.00 (14.59)	0%
Red and processed meat g/day (SD)	65.22 (33.20)	67.86 (34.47)	0%
Butter and hard margarines g/day (SD)	27.35 (23.09)	25.35 (22.64)	0%
Sugar and sweetened beverages g/day (SD)	126.45 (171.20)	156.19 (189.41)	0%
Total alcohol intake g/day (SD)	8.12 (9.69)	7.20 (8.93)	0%
Potatoes g/day (SD)	72.11 (52.20)	70.19 (52.38)	0%
Refined grain products g/day (SD)	74.24 (53.30)	80.76 (55.92)	0%
White unprocessed meat g/day (SD)	10.05 (7.80)	10.91 (8.20)	0%
Cheese g/day (SD)	32.45 (26.95)	29.92 (27.25)	0%
Savoury and ready products g/day (SD)	80.58 (60.65)	98.25 (67.17)	0%
Sugary products g/day (SD)	77.17 (49.55)	80.49 (50.68)	0%
Soups g/day (SD)	51.35 (54.11)	49.86 (52.65)	0%

Sweetened dairy g/day (SD)	97.17 (82.18)	101.28 (86.68)	0%
Artificially sweetened products g/day (SD)	72.22 (129.74)	81.14 (137.23)	0%
Vegetables g/day (SD)	104.07 (57.90)	102.90 (58.59)	0%
Fruits g/day (SD)	151.63 (119.35)	134.53 (110.69)	0%
Whole grain products g/day (SD)	105.59 (54.60)	113.06 (61.68)	0%
Legumes and nuts g/day (SD)	22.11 (22.00)	22.52 (23.18)	0%
Fish g/day (SD)	13.70 (13.49)	12.17 (12.65)	0%
Oils and soft margarines g/day (SD)	17.40 (17.53)	19.63 (19.00)	0%
Unsweetened dairy g/day (SD)	182.80 (166.86)	183.68 (165.06)	0%
Coffee g/day (SD)	448.72 (266.16)	415.68 (284.53)	0%
Tea g/day (SD)	246.82 (238.29)	243.62 (247.01)	0%
Total kilocalories (SD)	1986.02 (574.94)	2073 (624.26)	0%
Total kilojoules (SD)	8329.84 (2408.33)	8698.70 (2615.33)	0%
Total protein	73.51 (19.68)	75.52 (20.90)	0%
Plant based protein	30.02 (9.79)	31.71 (10.61)	0%
Animal based protein	43.60 (13.94)	43.91 (14.31)	0%
Total fat	78.68 (28.07)	82.30 (29.98)	0%
Total carbohydrates	219.79 (69.55)	233.01 (74.93)	0%
Total monosaccharaides	95.27 (40.13)	100.01 (42.24)	0%
Total polysaccharides	124.54 (41.14)	133.01 (44.59)	0%
Total free sugar	54.57 (35.13)	60.73 (37.85)	0%
Total added sugar	48.78 (32.28)	54.09 (34.74)	0%
Total glucose	11.56 (5.74)	12.19 (6.08)	0%
Total fructose	14.49 (7.32)	14.73 (7.35)	0%
Total lactose	13.46 (8.38)	13.70 (8.46)	0%
Total maltose	3.48 (1.57)	3.71 (1.66)	0%
Total sucrose	46.35 (26.79)	49.74 (28.19)	0%
Glycaemic index	584.50 (219.55)	616.81 (238.88)	0%
Glycaemic load	125.33 (41.86)	132.84 (44.89)	0%

*Body mass index was included, and not separately as weight and height due to multicolinearity; § Variables with more than 30% of missing data excluded from the analysis.

Table S2. Area under the receiver operator curve (AUC) for the cancer case-control models including all types of cancer and age groups.

Category	Model	All variables	Elastic net variable selection	Random forest variable selection
Matched case controls general	Random forest	0.5361 (0.5123-0.5598)	0.5573 (0.5337-0.5810)	0.5219 (0.4981-0.5457)
(matched by age, sex and educational level)	Logistic regression	0.5437 (0.5200-0.5675)	0.5591 (0.5355-0.5827)	0.5396 (0.5159-0.5633)
n = 8 464	Support vector machines	0.5414 (0.5176-0.5651)	0.5573 (0.5338-0.5808)	0.5361 (0.5123-0.5598)
Random case controls general	Random forest	0.6483 (0.6256-0.6711)	0.6489 (0.6262-0.6717)	0.6507 (0.6280-0.6734)
	Logistic regression	0.6436 (0.6208-0.6665)	0.6572 (0.6346-0.6798)	0.6613 (0.6388-0.6839)
n = 8 464	Support vector machines	0.6383 (0.6154-0.6612)	0.6454 (0.6322-0.6775)	0.6548 (0.6322-0.6775)
Case controls age group 1 (18-39)	Random forest	0.6260 (0.5672-0.6847)	0.5458 (0.4854-0.6062)	0.6107 (0.5514-0.6699)
	Logistic regression	0.5611 (0.5014-0.6207)	0.5573 (0.4970-0.6175)	0.5687 (0.5093-0.6281)
n = 1 314	Support vector machines	0.5344 (0.4744-0.5943)	0.5725 (0.5126-0.6325)	0.5534 (0.4945-0.6123)

Case controls age group 2 (40-49)	Random forest	0.5109 (0.4652-0.5565)	0.5457 (0.5001-0.5912)	0.5174 (0.4717-0.5631)
	Logistic regression	0.5261 (0.4804-0.5718)	0.5391 (0.4935-0.5847)	0.5239 (0.4782-0.5696)
n = 2 302	Support vector machines	0.5196 (0.4738-0.5653)	0.5391 (0.4940-0.5843)	0.5283 (0.4826-0.5740)
Case controls age group 3 (50-59)	Random forest	0.5430 (0.4923-0.5938)	0.5538 (0.5031-0.6044)	0.5538 (0.5031-0.6044)
	Logistic regression	0.5806 (0.5304-0.6309)	0.5726 (0.5231-0.6221)	0.5753 (0.5250-0.6256)
n = 1 864	Support vector machines	0.5511 (0.5004-0.6017)	0.5699 (0.5206-0.6191)	0.5457 (0.4951-0.5963)
Case controls age group 4 (60-69)	Random forest	0.5220 (0.4760-0.5680)	0.4890 (0.4429-0.5350)	0.5330 (0.4871-0.5790)
	Logistic regression	0.5176 (0.4719-0.5633)	0.5463 (0.5004-0.5921)	0.5088 (0.4628-0.5548)
n = 2 270	Support vector machines	0.5352 (0.4895-0.5810)	0.5441 (0.4988-0.5893)	0.5352 (0.4893-0.5812)
Case controls age group 5 (70+)	Random forest	0.5070 (0.4245-0.5896)	-	0.4718 (0.3895-0.5541)
	Logistic regression	0.4859 (0.4035-0.5683)	-	0.5282 (0.4456-0.6108)
n = 714	Support vector machines	0.5141 (0.4320-0.5962)	-	0.5352 (0.4526-0.6178)

Table S3. Area under the receiver operator curve (AUC) for the cancer case-control models breast, age groups breast.

Category	Model	All variables	Elastic net variable selection	Random forest variable selection
Breast case controls (matched by age and educational level)	Random forest	0.5076 (0.4581-0.5571)	0.4924 (0.4430-0.5418)	0.4949 (0.4455-0.5443)
	Logistic regression	0.4898 (0.4405-0.5392)	0.5127 (0.4653-0.5601)	0.5102 (0.4607-0.5596)
n = 1 974	Support vector machines	0.4884 (0.4354-0.5341)	0.5127 (0.4653-0.5601)	0.5051 (0.4556-0.5545)
Breast random controls	Random forest	0.5788 (0.5301-0.6275)	-	0.6195 (0.5272-0.6669)
	Logistic regression	0.5868 (0.5380-0.6357)	-	0.6123 (0.5640-0.6606)
n = 1 964	Support vector machines	0.5893 (0.5405-0.6381)	-	0.6020 (0.5535-0.6506)
Breast age group 1 (18-39)	Random forest	0.6364 (0.4935-0.7792)	0.5455 (0.3955-0.6954)	0.6136 (0.4665-0.7607)
	Logistic regression	0.5227 (0.3731-0.6724)	0.5455 (0.3955-0.6954)	0.4773 (0.3341-0.6205)
n = 226	Support vector machines	0.5909 (0.4480-0.7338)	0.5455 (0.3955-0.6954)	0.4773 (0.3341-0.6205)
Breast age group 2 (40-49)	Random forest	0.5127 (0.4342-0.5911)	-	0.5127 (0.4343-0.5910)
	Logistic regression	0.5506 (0.4726-0.6287)	-	0.5000 (0.4217-0.5783)
n = 794	Support vector machines	0.4873 (0.4091-0.5656)	-	0.4937 (0.4152-0.5721)
Breast age group 3 (50-59)	Random forest	0.5319 (0.4308-0.6330)	-	0.5426 (0.4408-0.6443)
	Logistic regression	0.4787 (0.3806-0.5768)	-	0.5532 (0.4524-0.6539)
n = 478	Support vector machines	0.5426 (0.4410-0.6441)	-	0.5532 (0.4517-0.6547)
Breast age group 4 (60-69)	Random forest	0.5513 (0.4395-0.6631)	-	0.5385 (0.4264-0.6505)
	Logistic regression	0.4744 (0.3651-0.5836)	-	0.4103 (0.3021-0.5184)
n = 390	Support vector machines	0.4744 (0.3639-0.5848)	-	0.5769 (0.4662-0.6877)
Breast age group 5 (70+)	Random forest	0.6875 (0.4469-0.9281)	-	0.6875 (0.4469-0.9281)

	Logistic regression	0.5625 (0.3047-0.8203)	-	0.5625 (0.3047-0.8203)
n = 86	Support vector machines	0.4375 (0.1969-0.6781)	-	0.5625 (0.3047-0.8203)

Table S4. Area under the receiver operator curve (AUC) for the cancer case-control models gastrointestinal, age groups gastrointestinal.

Category	Model	All variables	Elastic net variable selection	Random forest variable selection
Gastrointestinal case controls	Random forest	0.6033 (0.5416-0.6650)	0.5702 (0.5079-0.6326)	0.5909 (0.5293-0.6525)
(matched by age, sex and educational level)	Logistic regression	0.5661 (0.5036-0.6286)	0.5496 (0.4868-0.6124)	0.5537 (0.4913-0.6162)
n = 1218	Support vector machines	0.5537 (0.4908-0.6166)	0.5496 (0.4867-0.6125)	0.5909 (0.5290-0.6528)
Gastrointestinal random controls	Random forest	0.7355 (0.6804-0.7907)	-	0.7149 (0.6588-0.7709)
	Logistic regression	0.7231 (0.6666-0.7797)	-	0.7521 (0.6977-0.8064)
n = 1218	Support vector machines	0.7355 (0.6798-0.7913)	-	0.7231 (0.6667-0.7796)
Gastrointestinal age group 1 (18-39)	Random forest	-	-	-
	Logistic regression	-	-	-
n = 54	Support vector machines	-	-	-
Gastrointestinal age group 2 (40-49)	Random forest	0.5172 (0.3867-0.6478)	0.5345 (0.4045-0.6644)	0.6552 (0.5310-0.7793)
	Logistic regression	0.5172 (0.3864-0.6481)	0.5862 (0.4585-0.7139)	0.4828 (0.3522-0.6133)
n = 292	Support vector machines	0.5517 (0.4218-0.6817)	0.5172 (0.3867-0.6478)	0.5690 (0.4393-0.6986)
Gastrointestinal age group 3 (50-59)	Random forest	0.5000 (0.3716-0.6284)	-	0.5833 (0.4571-0.7096)
	Logistic regression	0.4833 (0.3554-0.6113)	-	0.5000 (0.3716-0.6284)
n = 300	Support vector machines	0.5000 (0.3739-0.6261)	-	0.5333 (0.4049-0.6617)
Gastrointestinal age group 4 (60-69)	Random forest	0.5833 (0.4769-0.6898)	-	0.5000 (0.3919-0.6081)
	Logistic regression	0.5476 (0.4400-0.6552)	-	0.5000 (0.3929-0.6071)
n = 422	Support vector machines	0.5119 (0.4040-0.6198)	-	0.5000 (0.3929-0.6071)
Gastrointestinal age group 5 (70+)	Random forest	0.6333 (0.4553-0.8114)	-	0.6000 (0.4202-0.7798)
	Logistic regression	0.7333 (0.5714-0.8953)	-	0.3667 (0.2105-0.5228)
n = 150	Support vector machines	0.6667 (0.4992-0.8341)	-	0.4333 (0.2536-0.6131)

Table S5. Area under the receiver operator curve (AUC) for the cancer case-control models prostate, age groups prostate.

Category	Model	All variables	Elastic net variable selection	Random forest variable selection
Prostate case controls (matched by age and educational level)	Random forest	0.5495 (0.4808-0.6182)	-	0.5198 (0.4513-0.5883)
	Logistic regression	0.5000 (0.4307-0.5693)	-	0.5099 (0.4409-0.5789)
n = 1 017	Support vector machines	0.5149 (0.4476-0.5821)	-	0.5149 (0.4457-0.5840)
Prostate random controls	Random forest	**0.8267** (0.7763-0.8772)	-	**0.8218** (0.7709-0.8726)
	Logistic regression	**0.7673** (0.7095-0.8252)	-	**0.8168** (0.7642-0.8694)
n = 1 017	Support vector machines	**0.8020** (0.7485-0.8555)	-	**0.8366** (0.7864-0.8868)
Prostate age group 1 (18-39)	Random forest	-	-	-
	Logistic regression	-	-	-
n = 5	Support vector machines	-	-	-
Prostate age group 2 (40-49)	Random forest	-	-	-
	Logistic regression	-	-	-
n = 50	Support vector machines	-	-	-
Prostate age group 3 (50-59)	Random forest	0.4600 (0.3247-0.5953)	-	0.5600 (0.4214-0.6986)
	Logistic regression	0.6200 (0.4828-0.7572)	-	0.5400 (0.3991-0.6809)
n = 256	Support vector machines	0.4600 (0.3285-0.5915)	-	0.5200 (0.3805-0.6595)
Prostate age group 4 (60-69)	Random forest	0.5283 (0.4324-0.6242)	-	0.5000 (0.4039-0.5961)
	Logistic regression	0.5283 (0.4330-0.6236)	-	0.5566 (0.4625-0.6507)
n = 534	Support vector machines	0.4623 (0.3673-0.5572)	-	0.5566 (0.4613-0.6519)
Prostate age group 5 (70+)	Random forest	0.7353 (0.5855-0.8851)	-	0.6471 (0.4815-0.8126)
	Logistic regression	0.5588 (0.3871-0.7306)	-	0.5000 (0.3344-0.6656)
n = 172	Support vector machines	0.4412 (0.2719-0.6105)	-	0.4706 (0.3025-0.6387)

File S1. About the chosen machine learning algorithms.

Three different algorithms were selected predict incident cancers, support vector machines, logistic regression and random forest[1,2]. Considering that classification (prediction) approaches rely either on probabilistic functions or merely dichotomous distinction

to assign binary labels, logistic regression and random forest belong to probabilistic class and support vector machines to the dichotomous distinction class[3].

Logistic regression:

Logistic regression is a widely used supervised model for dichotomous classification outcomes, either for univariate or multivariable analyses, the model works based on logistic function which discriminates between two categories built on a linear regression model calculated from the covariates. Logistic regression model assumes linearity, independence between observations and minimal or no multicolinearity among the independent variables.

Support vector machines:

Support vector machines is also a commonly used supervised algorithm for binary classification outcome. The model assumes that the input variables are independent, identically distributed and makes no assumptions over the underlying distributions of the independent variables. This model separates the input vectors by maximizing the edges of hyperplanes, also results effective when using high dimensional data [4].

Random forest:

Random forest is one of the most common used non-linear machine learning algorithms, growing trees depending on a random selection of input variables (continuous or dichotomous) decreasing the generalization error. No distribution assumptions are made for the input data, the final predictor is calculated by averaging the outcome of the k number trees [5].

References

1. Cruz, J.A.; Wishart, D.S. Applications of Machine Learning in Cancer Prediction and Prognosis. *Cancer Informatics* **2006**, *2*, 59–77, doi:10.1177/117693510600200030.
2. Kourou, K.; Exarchos, T.P.; Exarchos, K.P.; Karamouzis, M.V.; Fotiadis, D.I. Machine learning applications in cancer prognosis and prediction. *Comput. Struct. Biotechnol. J.* **2015**, *13*, 8–17, doi:10.1016/j.csbj.2014.11.005.
3. Dreiseitl, S.; Ohno-Machado, L. Logistic regression and artificial neural network classification models: a methodology review. *J. Biomed. Informatics* **2002**, *35*, 352–359, doi:10.1016/s1532-0464(03)00034-0.
4. Kecman, V. Support Vector Machines – An Introduction. *Flows in Networks Under Fuzzy Conditions* 2005, 1–47.
5. L. Breiman, Random Forests, Mach. Learn. (2001) 5–32. https://doi.org/10.14923/transinfj.2015IUP0008.
